# Explaining Entrepreneurial Status and Success from Personality: An Individual-Level Application of the Entrepreneurial Orientation Framework

**DOI:** 10.5334/pb.be

**Published:** 2015-06-04

**Authors:** Tim Vantilborgh, Jeroen Joly, Roland Pepermans

**Affiliations:** 1 Vrije Universiteit Brussel, Work & Organizational Psychology (WOPs), Belgium; 2 University of Toronto, Department of Political Science, Canada & Universiteit Antwerpen, Department of Political Sciences, Belgium

**Keywords:** Entrepreneurship, Entrepreneurial Orientation, Venture growth, Personality, Entrepreneurial status

## Abstract

Entrepreneurial orientation is defined as an organization’s strategy, describing its innovativeness, proactivity, risk taking, autonomy and competitiveness. We argue that this concept can be translated to the individual level as a constellation of five personality traits that characterize entrepreneurs. We examine the usefulness of these five traits in explaining entrepreneurial status and success. Our results show that entrepreneurs score higher than non-entrepreneurs on innovativeness, proactivity, and risk taking. In addition, latent growth curve modeling revealed that the individual EO traits were related to objective venture performance, albeit only after introducing venture life cycle as a moderator. In line with a differentiation perspective, risk taking, innovativeness, need for achievement, and need for autonomy were positively related to revenue and number of employees when venture life cycle was high. In line with a situation strength perspective, need for autonomy was positively related with growth in number of employees when venture life cycle was low. We conclude that individual entrepreneurial orientation offers a useful framework to understanding entrepreneurship once situational factors, such as venture life cycle, are taken into consideration.

## Introduction

The field of entrepreneurship has flourished in the past decades. It seems imperative to understand what drives people to become successful entrepreneurs, because entrepreneurs play a major role in economic development (Brandstätter, 2011). Many researchers therefore focused on the entrepreneurial orientation (EO) of firms. This construct refers to the strategies, processes, and styles of firms (Lumpkin & Dess, 1996). A firm that is entrepreneurially oriented will act autonomously, be innovative, take risks, take a competitive stance towards competitors, and proactively pursue new opportunities. Several studies have indeed shown that firms with a strong EO perform better than firms with a weak EO (e.g., Wiklund & Shepherd, 2005). While EO was originally considered a firm-level characteristic, it has recently been argued that EO can be translated to the individual level (Kollmann, Christofor, & Kuckertz, 2007; Krauss, Frese, Friedrich, & Unger, 2005). Yet, it remains unclear which traits would underly EO at the individual level and, more importantly, how EO at the individual level would relate to entrepreneurial status (i.e., being an entrepreneur or not) and to entrepreneurial success. Our goal is to address this gap in the literature, by empirically examining how individual EO relates to entrepreneurial status and success.

We define individual EO as an entrepreneurs’ tendency to desire autonomy, take risks, be innovative, take a competitive stance, and be proactive (Kollmann et al., 2007). It hence forms a constelation of five individual personality traits that drives entrepreneurial behavior (Kollmann et al., 2007). We make three major contributions to the literature. First, we focus on all five personality traits that form individual EO concomittantly. Although these five traits have been the subject of several studies on entrepreneurial personality, they have rarely been studied simultaneously. This nonetheless seems important, because the five traits likely relate to each other and share variance. Therefore, studying all five traits simultaneously is necessary to unravel the unique role of each trait. Second, we introduce venture life cycle as a moderator in the relationship between individual EO and entrepreneurial success. This is important because prior findings on the relationships between entrepreneurs’ personality traits and venture performance have often been inconsistent (Rauch & Frese, 2007). Some studies found that personality traits indeed related to venture performance (e.g., Rauch & Frese, 2007), whereas others found no statistically significant relationships (e.g., Baum & Locke, 2004; Gartner, 1989). This inconsistency could be due to situational factors that constrain the relationships between personality traits and firm performance (Hmieleski & Baron, 2009; Wiklund & Shepherd, 2005). One such situational factor is venture life cycle, defined as the various phases ventures go through as they grow and mature (Lester, Parnell, & Carraher, 2003). We propose that the relationship between the five individual EO traits and firm performance depends on the venture life cycle. In other words, we propose that personality traits may predict entrepreneurial success, but not necessarily in all phases of the ventures’ life cycle. We hence acknowledge that researchers should incorporate factors at various levels: (1) the environment in which the organization is active, (2) the organization itself, (3) the process or actions taken by individuals to start a new venture, and (4) the individual (e.g., the personality of the entrepreneur) (Kollmann, et al., 2007). These factors may interact, meaning that the influence of individual characteristics may be moderated by factors at other levels, such as the organization or the environment. Third, we focus on objective indicators of entrepreneurial success, namely growth in the amount of employees and growth in revenue over a period of three years. We hence avoid issues associated with subjective indicators of entrepreneurial success, such as common method bias (Podsakoff, MacKenzie, Lee, & Podsakoff, 2003).

### Individual entrepreneurial orientation

Previous research has given ample attention to entrepreneurs’ personality. Not surprisingly, the five dimensions of individual EO have already been used as personality traits to explain entrepreneurial behavior, albeit most often separately. Therefore, we will discuss each of the dimensions of individual EO and their relationship with entrepreneurial status and success separately. We follow the recommendation by Kollmann and colleagues (2007) and operationalize individual EO as five personality traits: innovativeness, proactivity, risk taking, need for autonomy, and need for achievement. We operationalize entrepreneurial status as being active as an entrepreneur or not. Entrepreneurial success is operationalized as revenue growth and employment growth (Murphy, et al., 1996). We develop hypotheses relating personality traits to entrepreneurial status and success, based on person-environment fit theory (Caplan, 1987) and the attraction-selection-attrition model (Schneider, 1987). According to these models, people become an entrepreneur because it matches their personality (Niess & Biemann, 2014). Moreover, these models suggest that some entrepreneurs will be more successful because their personality matches the activities and the entrepreneurial role and environment.

#### Innovativeness

Innovativeness is one of the oldest traits associated with entrepreneurs, reflecting a person’s willingness to create and develop new products and processes (Schumpeter & Opies, 1983). It is important to notice that innovation differs from creativity. Whereas creativity implies creating novel ideas, innovation also entails implementing or adapting novel ideas (Cromie, 2000). Given that innovation and entrepreneurship go hand-in-hand, it is not surprising that entrepreneurs are more innovative than non-entrepreneurs (Koh, 1996). In addition, entrepreneurs score higher on creativity tests than most other occupational groups, with the exception of teachers, lecturers and trainers (Cromie, 2000). While innovativeness can stimulate people to pursue an entrepreneurial career, hence acting as a motivator (Shane, Kolvereid, & Westhead, 1991), it can also enable entrepreneurs to perform better, thus acting as a facilitator. Successful entrepreneurs tend to more fluently produce original ideas than unsuccessful entrepreneurs (Ames & Runco, 2005). This can help them come up with new venture opportunities or devise alternative solutions to existing problems, hence increasing venture performance. A meta-analysis by Rauch & Frese (2007) indeed demonstrated that innovativeness positively correlated with venture success. We therefore propose:

*Hypothesis 1a: Entrepreneurs score higher on innovativeness than non-entrepreneurs.*
*Hypothesis 1b: Innovativeness positively relates to entrepreneurial success.*

#### Proactivity

Entrepreneurs start their own business by seizing an opportunity. Hence they are very likely to display a proactive personality: a tendency to influence their environment by identifying opportunities and acting on them, showing initiative, taking action, and persevering until meaningful change occurs (Crant, 2000). Moreover, high proactive individuals have higher entrepreneurial intentions (Crant, 1996). A recent meta-analytic study also established proactivity as an important predictor of venture success (Rauch & Frese, 2007). This comes as no surprise as proactivity has been associated with both objective and subjective career success, in a diverse set of occupations and organizations (Seibert, Kraimer, & Crant, 2001). Proactivity might influence entrepreneurial success through the strategy chosen by the entrepreneur. For instance, proactive entrepreneurs tend to adopt a prospector strategy, intensively scanning their environment for new opportunities, and focusing on product development and market research (Kickul & Gundry, 2002). This in turn might give them an edge over competitors who adopt other strategies. Hence, we propose:

*Hypothesis 2a: Entrepreneurs score higher on proactivity than non-entrepreneurs.*
*Hypothesis 2b: Proactivity positively relates to entrepreneurial success.*

#### Risk Taking

As entrepreneurs function in an uncertain, unstructured environment, bearing ultimate responsibility for their decisions, they inevitably become exposed to a certain amount of risk. Consequently, several authors claim entrepreneurs are more risk-tolerant and differ from non-entrepreneurs in their propensity towards risks (Perry, 1990; Rauch & Frese, 2007; Stewart & Roth, 2001). Indeed, Niess and Biemann (2014) demonstrated in a longitudinal study that people who are risk tolerant are more likely to become self-employed. However, entrepreneurs are no gamblers: they take calculated risks (Stewart & Roth, 2001). Moreover, a business opportunity might appear risky to outsiders but not to experienced entrepreneurs, as the latter possess more accurate knowledge and information to appropriately evaluate its risk (Chell, Haworth, & Brearley, 1991). This ability to properly judge and subsequently act, where others would not, on risky but rewarding opportunities can stimulate business growth and create a competitive advantage. Indeed, Krauss and colleagues (2005) demonstrated that entrepreneur’s risk taking explained business growth, although this effect disappeared when they added additional personality traits to their model. Interestingly, Niess and Biemann (2014) showed that entrepreneurs with medium risk taking levels were most likely to remain as an entrepreneur. This suggests the potential existence of a curvilinear relationship between risk taking and entrepreneurial success, as taking too much risk may actually be detrimental for business survival. We nonetheless propose and test a positive linear effect of risk taking on entrepreneurial success, given that Rauch and Frese’s (2007) meta-analysis supported the presence of such a linear relationship and because curvilinear relationships are not the main focus of our study.

*Hypothesis 3a: Entrepreneurs score higher on risk taking than non-entrepreneurs.*
*Hypothesis 3b: Risk taking positively relates to entrepreneurial success.*

#### Need for autonomy

People with a strong need for autonomy desire to have a high degree of autonomy in various aspects of their lives (Kollmann et al., 2007), which implies that they are motivated to independently take decisions and have control (Perry, 1990). Because people differ from each other in their need for autonomy, it can be considered an individual difference that is often studied from a trait perspective (e.g., Brandstätter, 2011). Entrepreneurs strive for control: they try to avoid restrictive environments (rules, procedures, social norms) and display a higher need for autonomy than other occupational groups (Cromie & O’Donaghue, 1992). Need for autonomy is also a frequently mentioned key motive for starting a venture (Perry, 1990). However, there have been fewer studies that related need for autonomy to venture success. Overall, it has been argued that autonomous individuals may achieve their goals faster because their environment hinders them less. Perry (1990) indeed found that independence differentiated successful from failed entrepreneurs. Moreover, Rauch and Frese’s (2007) meta-analysis found a positive relationship between need for autonomy and venture performance.

*Hypothesis 4a: Entrepreneurs score higher on need for autonomy than non-entrepreneurs.*
*Hypothesis 4b: Need for autonomy positively relates to entrepreneurial success.*

#### Need for achievement

Not surprisingly, (global) competition forces entrepreneurs to take an aggressive stance towards rivals. Competitive aggressiveness indicates that entrepreneurs have a combative attitude and attempt to outperform business rivals (Lumpkin & Dess, 2001). From this point of view, there is a clear link between competitive aggressiveness and need for achievement^1^ (Kollmann, et al., 2007). People scoring high on need for achievement are more likely to “engage in energetic and innovative activities that require planning for the future and entail an individual’s responsibility for task outcomes” (Collins, Hanges & Locke, 2004, p. 96). Like need for autonomy, need for achievement can be considered an individual difference that is often studied from a trait perspective (e.g., Brandstätter, 2011). People with a high need for achievement also prefer tasks that require skill and effort, are challenging and provide immediate feedback. Hence, McClelland already suggested in 1967 that these people would be attracted to and perform well in entrepreneurial jobs. A recent meta-analytic study supported that need for achievement differentiates between entrepreneurs and non-entrepreneurs, although there were few differences between managers and entrepreneurs (Collins, et al., 2004). The characteristics of managerial jobs may also attract high need for achievement individuals (Cromie, 2000). The aforementioned meta-analysis also revealed that need for achievement differentiated successful from unsuccessful entrepreneurs (Collins, et al., 2004). Similarly, Krauss and colleagues (2005) demonstrated that need for achievement explained business growth, even when controlling for other personality traits. Hence, we propose that:

*Hypothesis 5a: Entrepreneurs score higher on need for achievement than non-entrepreneurs.*
*Hypothesis 5b: Need for achievement positively relates to entrepreneurial success.*

### The moderating role of venture life cycle

Previous studies on the relationships between entrepreneurs’ personality traits and entrepreneurial success often led to inconclusive findings (Baum & Locke, 2004; Gartner, 1989). For example, Rauch and Frese’s (2007) meta-analysis demonstrated that innovativeness, proactivity, risk taking, need for autonomy, and need for achievement were positively related to venture performance. However, other scholars failed to find statistically significant relationships between one or more of these five personality traits and various indicators of entrepreneurial success (e.g., Begley & Boyd, 1987; Kraus et al., 2005; Zhao et al., 2010). It has therefore been suggested that moderators need to be taken into account to explain these inconsistent findings (Hmieleski & Baron, 2009). We therefore focus on venture life cycle as a moderator in the relationship between individual EO and entrepreneurial success. The underlying reason is that leaders require distinct characteristics along various stages of the venture’s life cycle (Hmieleski & Baron, 2009). Smith, Mitchell, and Summer (1985) distinguished three distinct life-cycle stages. First, organizational inception and mobilization refers to gathering resources and getting the organization going. Second, growth refers to expanding the organization. Third, maturity refers to managing a status quo or restructuring for new growth. Based on the literature, we contrast two opposing perspectives on the potential moderating role of venture life cycle: a situation strength perspective and a differentiation perspective.

Based on *the situation strength perspective*, various venture life cycles may be characterized by different levels of situation strength, defined as “implicit or explicit cues provided by external entities regarding the desirability of potential behaviors” (Meyer, Dalal, & Hermida, 2010, p. 122). According to this perspective, one’s personality will exert a stronger influence on one’s behavior in weak, ambiguous situations. In contrast, strong situations have clear cues that signal how people should behave, meaning that personality will exert a weaker influence on one’s behavior. It has been suggested that as organizations mature, they formalize their management and practices (Mintzberg, 1998), leaving less room for the entrepreneur to influence organizational processes. In other words, later venture life cycles may represent strong situations, whereas early venture life cycles could be considered weak situations. Organizations in an early life cycle operate in an uncertain, dynamic environment, lacking cues on the desirability of entrepreneurial behavior. Because these constitute “weak” situations, personality traits ought to have a strong effect on entrepreneurial behavior. In contrast, organizations in a mature life cycle are formalized and hence can be considered strong situations, possessing clear guidelines on how one ought to behave. In such situations, personality traits ought to have weaker effects on entrepreneurial behavior.

In contrast, *the differentiation perspective* suggests that an entrepreneur’s personality may offer a competitive advantage in certain phases of the venture life cycle. As organizations enter successive life cycles, isomorphism will cause them to gradually become more similar (Powell & DiMaggio, 2012). For organizations in a later venture life cycle, the entrepreneur’s personality may help to differentiate the firm from other, similar organizations and, hence, offer a unique competitive advantage. Indeed, Wiklund and Shepherd (2005) demonstrated that firm-level EO had a stronger positive relationship with firm performance in stable environments, compared to dynamic environments. Based on these findings, one might argue that individual EO has a stronger relationship with firm performance as venture life cycle increases.

In sum, we propose that venture life cycle will moderate the relationship between individual EO and firm performance. Hence, we take an exploratory stance and do not specify a direction for this moderator effect, because the situation strength and the differentiation perspectives suggest opposite effects. Whereas the situation strength perspective suggests that individual EO is more strongly related to entrepreneurial success in early venture life cycles, the differentiation perspective suggests that it is more strongly related in later venture life cycles.

Hypothesis 6. Venture’s life cycle moderates the effects of individual EO on entrepreneurial success.

## Method

### Sample

Three samples were gathered for this study. We started by collecting a pilot study sample to fine-tune our measures of individual EO (*N* = 202). This sample contained slightly more women (52%) than men (48%), and had a mean age of 32 years (*SD* = 10.91). Most respondents attained a master (46.4%) or bachelor degree (32.6%), while some only finished secondary (16.7%) or primary school (4.3%). This pilot study sample contained actual entrepreneurs (14.5%), nascent entrepreneurs (31.5%), and non-entrepreneurs (54%). Next, we collected two main study samples: a main study sample containing only entrepreneurs and a main study sample containing non-entrepreneurs. For the main study entrepreneur sample, 1710 Belgian registered venture owners were randomly selected from a database enveloping information on ventures (Trends, 2007). They were personally sent an email containing a link to our online survey, yielding a response rate of 21.7 %. However, to be included in the final main study entrepreneur sample, respondents had to be both founder and manager of a venture, which finally led to a sample of 218 entrepreneurs. This main study entrepreneur sample was predominantly male (88.5%) with an average age of 47 years (*SD* = 9.22). Respondents mostly attained a master (37.6%) or bachelor degree (37.6%), followed by secondary (19.7%) and primary school degrees (5%). While these demographic characteristics are comparable to previous studies (Delmar & Davidsson, 2000), our sample contained more males (χ²(1, *N* = 218) = 41.73, *p* < .001) and more 40 to 59 year old entrepreneurs (χ²(6, *N* = 218) = 73.43, *p* < .001) than the general Belgian entrepreneurial population. This stresses the importance of including age, gender, and education as control variables in further analyses. Our main study non-entrepreneur sample was intended as a control group and consisted of working employees (*N* = 772) instead of entrepreneurs. These respondents were first contacted through an online panel. This main study non-entrepreneur sample contained slightly more male (58%) respondents, while respondents had an average age of 45.01 years (*SD* = 11.45). Most respondents on the non-entrepreneurs sample had attained a bachelor (34.84%) or secondary school (30.70%) degree, followed by primary school (18.78%) and master (15.67%) degrees.

### Measures

#### Independent variables

We measured innovation with the innovation subscale of the *Jackson Personality inventory* (Jackson, 1994), using a six-point Likert scale ranging from “strongly disagree” (1) to “strongly agree” (6). A sample item was: “I enjoy thinking of original plans on which to work”. Proactivity was measured using the *Proactive Personality scale* by Bateman & Crant (1993). A sample item was: “If I believe in an idea, no obstacle will prevent me from making it happen”. We measured risk taking with the risk taking innovation subscale of the *Jackson Personality inventory* (Jackson, 1994), using a six-point Likert scale ranging from “strongly disagree” (1) to “strongly agree” (6). A sample item was: “Taking risks doesn’t bother me if the gains involved are high”. We measured need for autonomy using the autonomy subscale of the *Manifest Needs Questionnaire* by Steers and Braunstein (1976) and the autonomy subscale of the *Entrepreneurial Orientation Scale* by Hughes and Morgan (2007). Sample items were: “In my work assignments, I try to be my own boss” and “It is important to be able to act and think without interference” for both measures respectively. Finally, we measured need for achievement using the achievement subscale of the *Manifest Needs Questionnaire* by Steers and Braunstein (1976) and the *Achievement Motivation* scale by Othman, Ghazali, & Sung (2006). Sample items were: “I try very hard to improve on my past performance at work” and “I prefer to work in situations that require a high level of skills” for both measures respectively. All measures used a seven-point Likert scale ranging from “strongly disagree” (1) to “strongly agree” (7), unless otherwise noted. All items were back-translated into Dutch and French (the two major native languages in Belgium).

We had to address two issues regarding these measures before we could administer them. First, questionnaires intended for entrepreneurs should be developed for this particular population, taking into account the specific characteristics of entrepreneurial activity (Robinson, Stimpson, Huefner, & Hunt, 1991). However, with exception of the Entrepreneurial Orientation scale and the Achievement Motivation scale, the aforementioned measures target the general population. Hence, we had to revise certain items to optimize them for our sample of entrepreneurs. Second, the five dimensions of individual EO share certain similarities. Hence, it is possible that items measuring one dimension also tap into other dimensions, decreasing measurement validity. Therefore, we had to assess our measures’ dimensional structure and omit cross-loading variables, before applying them in our main study.

A pilot study was conducted to address these two issues. In the process of this pilot study, the original number of items got reduced from 77 to 43 items. The evaluation of scales and items in the pilot study took place in two subsequent phases, following recommendations for scale development by DeVellis (2003). In the first phase, five expert judges (academics active in the field of organizational behavior) evaluated all items on formulation (e.g. ambiguous wording, use of jargon) and face validity. In the second phase, the remaining items were sent as an online survey to the pilot study sample described earlier (*N* = 202). We evaluated scales using three criteria: (1) item variance, (2) factor structure, and (3) internal reliability. First, items scoring high on both kurtosis and skewness were deleted as they did not have enough variance. Second, as we had an a-priori factor structure (with five factors) that we wanted to test, we employed confirmatory factor analysis in Mplus version 7.11 (Muthén & Muthén, 2012). After inspecting the modification indices, we deleted items with high cross-loadings (> .40; Stevens, 2002) on multiple dimensions. We assessed the model fit of a five-factor model (i.e., a latent variable for each dimension of individual EO), based upon recommendations by Kline (2005). Model parameters were estimated using maximum likelihood, and stratification was used to account for the fact that the pilot-study sample contained a mix of entrepreneurs and non-entrepreneurs (Muthén & Muthén, 2012). The five-factor model attained a good fit to the data, except for the RMSEA indicator (χ² = 4390.34, df = 730, *p* < .001, recommended *p* > .05; RMSEA = .26, recommended < .06; CFI = .95, recommended > .90; TLI = .94, recommended > .90). Moreover, the five-factor model offered a significantly better fit (Δχ² = 7663.27, Δdf = 89, *p* < .001) to the data than a one-factor model (χ² = 12053.63, df = 819, *p* < .001, RMSEA = .42, CFI = .83, TLI = .82). We retested the factor structure in the main study samples (main study entrepreneur sample and main study non-entrepreneur sample combined); the five-factor model again offered a good fit to the data (χ² = 3670.74, df = 730, *p* < .001; RMSEA = .06; CFI = .99; TLI = .99). Moreover, the five-factor model offered a significantly better fit (Δχ² = 4895.37, Δdf = 89, p < .001) to our data than a one-factor model (χ² = 8566.11, df = 819, *p* < .001; RMSEA = .10; CFI = .97; TLI = .97).

Third, we tested internal reliability of each dimension using Cronbach alpha scores with a minimum criterion of .60. The end result of these steps was a questionnaire consisting of 43 items, measuring innovation (15 items), proactivity (7 items), risk taking (7 items), need for autonomy (6 items), and need for achievement (8 items).

#### Moderator variable

We focused on venture life cycle as a moderator. While some studies use subjective measures of venture life cycle by asking entrepreneurs to categorize their ventures into life cycle phases (e.g., Covin & Slevin, 1990), we opted for a measure that is based on three objective indicators: firm age, size in number of employees, and sales growth (Rutherford, Buller, & McMullen, 2003). Rutherford and colleagues (2003) argue that such an operationalization is preferred over subjective measures, because it does not impose a particular life cycle model on participants. They explain that there are vast differences between life cycle models. The amount of life cycle phases proposed by various models, for instance, ranges from 3 to 10. Therefore, they recommend deriving venture life cycle based on objective indicators. In contrast with Rutherford and colleagues (2003), we wanted to use venture life cycle as a continuous moderator, and therefore created a compound variable instead of using a cluster analysis technique to create categorical variables representing life cycle phases. In particular, we standardized data on firm age, size in number of employees and sales growth for the year prior to the data collection and averaged these standardized variables to create a measure of venture life cycle. Data on these three objective indicators were collected from the BEL-FIRST database (Bureau Van Dijk, 2007).

#### Control variables

Individual-level control variables included respondents’ gender (0 = male, 1 = female), age (in years), and education (1 = primary school, 2 = secondary school, 3 = bachelor degree, 4 = master degree, 5 = higher (than master) degrees). These variables were gathered as demographic items at the beginning of the questionnaire.

#### Dependent variables

Entrepreneurial status was defined as being active as an entrepreneur (no = 0, yes = 1) at the time of the survey. We defined entrepreneurs as the founders of a venture, who were currently also in charge of that venture. Entrepreneurs needed to provide their VAT-number proving they currently owned and managed the venture. Respondents who reached these criteria were labeled as active entrepreneurs. Entrepreneurial success was operationalized using revenue and employment data of the ventures over a three-year period prior to the survey administration. Both revenue and employment can be considered widespread indicators of venture performance (Murphy, et al., 1996). These data were obtained from the BEL-FIRST database (Bureau Van Dijk, 2007), which gathers venture’s financial information based on reports for each booking year.

### Procedure

Respondents from both the entrepreneur and the control sample were contacted through email. They received a brief overview of the research goals, and were guaranteed confidentiality in the introduction to the survey. In addition, we stated that there were no wrong answers and we asked respondents to answer truthfully. Results were gathered by the authors and analyzed in Mplus version 7.11 (Muthén & Muthén, 2012) and in R version 2.3 (R Core Team, 2013). Hierarchical logistic regression analysis was used to test differences between entrepreneurs and non-entrepreneurs, while latent growth curve modeling was used to test if individual EO was related to the intercept (i.e., level of the dependent variable during the first year) and the slope (i.e., linear change in the dependent variable) of revenue and number of employees over a three-year period (see Preacher, Wichman, MacCallum, & Briggs, 2008 for a detailed discussion of latent class growth modeling). The five individual EO traits, venture life cycle, and the interaction terms were entered as covariates in these latent growth curve models. Independent variables were mean-centered before creating the interaction terms (Cohen, Cohen, West, & Aiken, 2003). Simple slopes plots were later on used to interpret significant interaction effects.

## Results

### Descriptive statistics

Table 1 shows the descriptive statistics for the entire sample. Internal reliability estimates ranged from .67 to .87. As can be seen in Table 1, the five individual EO dimensions correlated positively with each other and with entrepreneurial status (being an entrepreneur (1) or not (0)). In addition, risk taking was positively correlated to revenue of entrepreneurs’ organizations for all three included years.

**Table 1:** Descriptive statistics (internal reliability estimates between brackets).

Variable	M	SD	1.	2.	3.	4.	5.	6.	7.	8.	9.	10.	11.	12.	13.	14.	15.

1. Age	45.55	11.04															
2. Gender	.65	.48	.20***														
3. Education	2.61	.98	−.07*	.07*													
4. Innovativeness	4.05	.65	.07*	.20***	.26***	(.87)											
5. Proactivity	5.13	.76	.11***	.24***	.21***	.66***	(.71)										
6. Risk taking	3.89	.76	.04	.25***	.21***	.50***	.49***	(.67)									
7. Need for autonomy	4.53	.93	.06	.07*	.07*	.21***	.26***	.30***	(.72)								
8. Need for achievement	5.11	.78	.11***	.19***	.21***	.52***	.68***	.51***	.32***	(.75)							
9. Entrepreneur status	.22	.41	.09**	.26***	.25***	.33***	.33***	.29***	.09**	.22***							
10. Venture life cycle	−.12	.70	.24***	.09	−.08	−.15*	−.12	.01	−.06	.02							
11. Revenue T1	10785.02	19182.07	.10	.14	−.09	−.06	−.11	.24*	.13	.10	.	.37**					
12. Revenue T2	7737.28	14126.56	.07	.12	−.05	−.08	−.16	.23*	.05	.09	.	.41***	.98***				
13. Revenue T3	7653.38	14319.25	.13	.11	−.07	−.12	−.18	.24*	.09	.14	.	.37***	.90***	.94***			
14. Employment T1	25.94	152.71	.14	.04	−.02	.06	.03	.08	.05	.07	.	.50***	.30*	.30**	.28*		
15. Employment T2	20.17	86.37	.15	.05	−.02	.07	.04	.10	.06	.08	.	.54***	.33*	.35**	.31**	.99***	
16. Employment T3	19.16	83.13	.15	.04	−.02	.05	.03	.09	.05	.07	.	.57***	.39*	.37**	.32**	.9***	1.00***

*Notes: N* = 990 (*N*(entrepreneurs) = 218, *N*(non−entrepreneurs) = 772). * p < .05, ** *p* < .01, *** *p* < .001.

### Explaining entrepreneurial status

Hypotheses 1a, 2a, 3a, 4a and 5a stated that the five dimensions of individual EO explain entrepreneurial status (being active as an entrepreneur (1) or not (0)). We performed a logistic regression analysis, in which we regressed entrepreneurial status on the control variables age, gender, and education, and the five individual EO variables (innovation, proactivity, risk taking, need for autonomy, need for achievement) (see Table 2). This model was statistically significant (χ²(8) = 239.70, *p* < .001) and explained between 21.62% (Cox and Snell R-squared) and 33.26% (Nagelkerke R-squared) of the total variance in entrepreneurial status. As shown in Table 2, innovativeness, proactivity, and risk taking were significantly positively related to entrepreneurial status. We thus could confirm hypotheses 1a, 2a and 3a. Of these three individual EO variables, proactivity was the strongest predictor of entrepreneurial status, recording an odds ratio of 2.17.

**Table 2:** Results of logistic regression analysis explaining entrepreneurial status.

Variable	Beta (SE)	95% CI for odds ratios		

		Lower	Odds ratio	Upper

Intercept	−11.47 (1.03)***	0.00	0.00	0.00
Age	0.01 (0.01)	0.99	1.01	1.03
Gender	1.38 (0.25)***	2.50	3.98	6.56
Education	0.53 (0.10)***	1.40	1.69	2.06
Innovativeness	0.63 (0.20)**	1.27	1.87	2.78
Proactivity	0.77 (0.19)***	1.49	2.17	3.18
Risk taking	0.49 (0.15)***	1.23	1.64	2.20
Need for autonomy	−0.02 (0.10)	0.81	0.98	1.19
Need for achievement	−0.29 (0.16)	0.55	0.75	1.02

*Notes: N* = 984, * p<.05, ** *p* <.01, *** *p*<.001.

### Explaining entrepreneurial success

Hypotheses 1b, 2b, 3b, 4b and 5b investigated whether the individual EO dimensions explained entrepreneurial success, captured by absolute differences between ventures (i.e., the intercepts of the latent growth curve models) and by linear change over time within a venture (i.e., the slope of the latent growth curve models) in terms of revenue and number of employees. In addition, hypothesis 6 stated that these effects would be moderated by the venture’s life cycle. We estimated two latent growth models, the one modeling growth in revenue and the other modeling growth in the number of employees. In each model, we fitted an intercept and slope function to the three-year spanning objective data on revenue and number of employees respectively. Next, we added covariates to explain variance in the intercept and the slope of these models. Below, we discuss both latent growth models separately.

#### Revenue

We started by fitting an intercept and slope latent growth model to revenue data for each firm over a period of three years. Revenue data were standardized prior to estimating the latent growth model, to avoid convergence issues. This latent growth model fitted the data well, except for the RMSEA indicator (χ^2^(2) = 12.59, *p* < .01, RMSEA = .24, CFI = .97, TLI = .95, SRMR = .04). Subsequently, we added the main effects of the covariates to the model. This main effects model offered a good fit to the data (χ^2^(11) = 20.07, *p* = .04, RMSEA = .09, CFI = .97, TLI = .93 SRMR = .01). Table 3 shows the estimates for the relationships between the covariates and the intercept and slope of revenue. As can be seen in Table 3, the covariates significantly explained 21% of the variance in the intercept and 22.2% of the variance in the slope of revenue. Venture life cycle was positively related to the intercept of revenue (β = .41, *z* = 3.69, *p* < .001). In contrast to our expectations, proactivity was negatively related to change (i.e., the slope) in revenue (β = ­.11, *z* = ­2.13, *p* = .03). The other individual EO variables were not significantly related to the intercept or the slope of revenue. Hence, we could not confirm Hypotheses 1b to 5b.

**Table 3:** Effects of covariates in latent growth model of revenue.

Covariates	Main effects model		Interaction model 1		Interaction model 2		Interaction model 3		Interaction model 4		Interaction model 5	

	Intercept	Slope	Intercept	Slope	Intercept	Slope	Intercept	Slope	Intercept	Slope	Intercept	Slope

Intercept							.29	−.20	.43	−.02	.19	−.01
Age	−.01	.002	−.01	.003	−.01	.002	−.01	.003	−.02	.002	−.01	.003
Gender	.28	.002	.29	−.001	.28	.003	.22	.002	.34	.01	.29	−.003
Education	−.05	−.02	−.04	−.03	−.05	−.02	−.01	−.02	−.01	−.02	−.02	−.03
Innovativeness (INN)	−.03	−.03	−.04	−.03	−.04	−.04	−.04	−.04	−.06	−.04	−.07	−.03
Proactivity (PRO)	−.24	−.11*	−.27	−.11*	−.24	−.11*	−.33	−.11*	−.24	−.11*	−.26	−.11*
Risk taking (RT)	.22	.03	.22	.03	.21	.03	.22	.03	.20	.03	.17	.04
Need for autonomy (AUT)	.10	.02	.10	.02	.10	.02	.11	.02	.12	.02	.10	.02
Need for achievement (ACH)	.04	.06	.04	.06	.04	.06	.001	.06	.04	.06	.02	.06
Venture life cycle (VLC)	.41***	.01	.41***	.02	.41***	.02	.31**	.01	.44***	.02	.35***	.02
INN x VLC			.14	−.04								
PRO x VLC					.03	.01						
RT x VLC							.43*	.001				
AUT x VLC									.31*	.02		
ACH x VLC											.42*	−.05

*R2*	.21**	.22*	.21**	.23*	.21**	.22*	.26***	.22*	.26***	.22*	.26***	.25*
BIC	308.35	310.34	311.06	304.67	305.00	305.10

*Notes: N* = 218, * p < .05, ** *p* < .01, *** *p* < .001.

Next, we examined if the relationships between the individual EO variables and the intercept and slope of revenue were moderated by venture life cycle. Therefore, we estimated five models in which we added the interaction between venture life cycle and the five individual EO variables separately. We compared the sample-size adjusted Bayesian Information Criterion (BIC) of each interaction model to that of the main effects model. Interaction effects were only interpreted when the BIC value of the interaction model was smaller than that of the main effects model. As can be seen in Table 3, the BIC values of the interaction models that included risk taking (interaction model 3), need for autonomy (interaction model 4), and need for achievement (interaction model 5) were smaller than the BIC value of the main effects model. As can be seen in these models, the interaction effects of risk taking, need for autonomy, and need for achievement with venture life cycle were significantly and positively related to the intercept of revenue.

**Figure 1:**
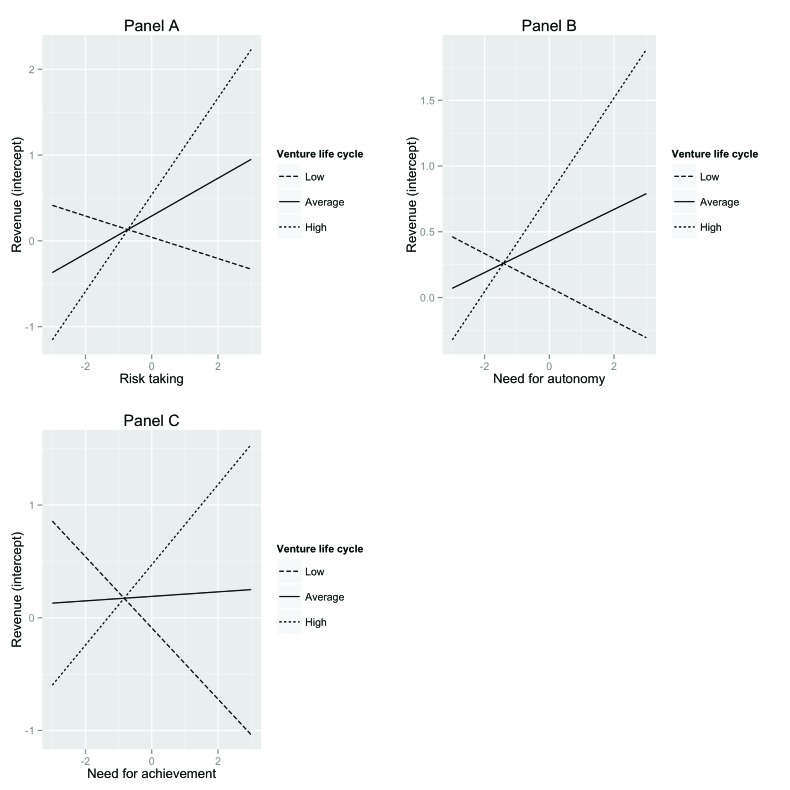
Simple slope plots for interaction effects of venture life cycle and risk taking (panel A), venture life cycle and need for autonomy (panel B), venture life cycle and need for achievement (panel C) explaining variance in the intercept of revenue.

We plotted simple slopes to interpret these three significant interaction effects (Preacher, Curran, & Bauer, 2006). Specifically, we plotted the relationship between the individual EO variable and the intercept of revenue for low (1SD below the mean), average, and high (1SD above the mean) values of venture life cycle. Figure 1 shows these simple slopes for the three significant interaction terms described above. As can be seen in Panel A of Figure 1, there is a significant positive relationship between risk taking and the intercept of revenue when venture life cycle is high (*est.* = .56, *t* = 3.09, *p* = .002), and non-significant relationships between risk taking and the intercept of revenue when venture life cycle is average (*est.* = .22, *t* = 1.79, *p* = .08) or low (*est.* = ­.12, *t* = ­.68, *p* = .50). Likewise in Panel B, there is a significant positive relationship between need for autonomy and risk taking when venture life cycle is high (*est.* = .37, *t* = 2.55, *p* = .01). The relationship between need for autonomy and the intercept of revenue was not significant for average (*est.* = .12, *t* = 1.38, *p* = .17) and low (*est.* = ­.13, *t* = ­.98, *p* = .33) values of venture life cycle. Panel C shows that there was a positive, albeit non-significant, relationship between need for achievement and the intercept of revenue when venture life cycle was high (*est.* = .36, *t* = 1.81, *p* = .07). This relationship became significant when venture life cycle was 1.23 standard deviations above the mean. The relationship between need for achievement and the intercept of revenue was not significant when venture life cycle was average (*est.* = .02, *t* = .14, *p* = .89) or low (*est.* = ­.32, *t* = ­1.52, *p* = .13). This negative relationship became significant when venture life cycle was 1.78 standard deviations below the mean. In sum, we could partly confirm Hypothesis 6, as venture life cycle moderated the relationships in three of the five individual EO variables and the intercept of revenue.

#### Number of employees

We again started by fitting an intercept and slope latent growth model to the employment data for each firm over a period of three years. Employment data were standardized prior to estimating the latent growth model, to avoid convergence issues. This latent growth model fitted the data well (χ^2^(2) = 4.80, *p* = .09, RMSEA = .09, CFI = 1.00, TLI = 1.00, SRMR = .05). Subsequently, we added the main effects of the covariates to the model. This main effects model offered a good fit to the data (χ^2^(11) = 7.11, *p* = .79, RMSEA = .00, CFI = 1.00, TLI = 1.00, SRMR = .02). Table 4 shows the estimates for the relationships between the covariates and the intercept and slope of revenue. As can be seen in Table 4, the covariates significantly explained 31.4% of the variance in the intercept, but did not explain a significant amount of variance (1%) in the slope of number of employees. Venture life cycle was positively related to the intercept of employment (β = .30, *z* = 8.53, *p*<.001). The other individual EO variables were not significantly related to the intercept of employment. Hence, we could not confirm Hypotheses 1b to 5b.

**Table 4:** Effects of covariates in Latent Growth Model of number of employees.

Covariates	Main effects model		Interaction model 1		Interaction model 2		Interaction model 3		Interaction model 4		Interaction model 5	

	Intercept	Slope	Intercept	Slope	Intercept	Slope	Intercept	Slope	Intercept	Slope	Intercept	Slope

Intercept	.02	.01	.01	.01	.03	.01	−.01	.01	.05	.01	.03	.01
Age	−.001	.00	−.001	.00	−.001	.00	−.001	.00	−.002	.00	−.002	.00
Gender	.02	.02	−.01	.02	.01	.02	.004	.02	.01	.02	.02	.02
Education	−.01	−.01	.001	−.01	−.004	−.004	.01	−.01	.01	−.01	.004	−.01
Innovativeness (INN)	.08	−.01	.09	−.01	.08	−.01	.08	−.01	.07	−.01	.07	−.002
Proactivity (PRO)	.02	.01	−.004	.01	.02	.004	−.01	.01	.03	.004	.02	.004
Risk taking (RT)	.02	.01	.02	.01	.01	.01	.02	.01	.01	.01	.001	.01
Need for autonomy (AUT)	.03	−.001	.03	−.001	.03	−.001	.03	−.001	.03	−.001	.02	−.001
Need for achievement (ACH)	−.004	−.004	−.003	−.004	−.003	−.004	−.01	−.003	−.01	−.004	−.01	−.001
Venture life cycle (VLC)	.30***	.01	.29***	.01	.30***	.01	.26***	.02	.30***	.01	.27***	.02
INN x VLC			.22***	−.02								
PRO x VLC					.07	.02						
RT x VLC							.18***	−.03				
AUT x VLC									.17***	−.002		
ACH x VLC											.27***	−.05***

*R^2^*	.31***	.03	.36***	.04	.32***	.04	.36***	.05	.37***	.03	.39***	.10*
BIC	−379.67	−389.22	−378.02	−388.92	−390.00	−407.78

*Notes: N* = 218, * p < .05, ** *p* < .01, *** *p* < .001.

Next, we examined if the relationships between the individual EO variables and the intercept and slope of number of employees were moderated by venture life cycle. Therefore, we estimated five models in which we added the interaction between venture life cycle and the five individual EO variables separately. We again compared the sample-size adjusted Bayesian Information Criterion (BIC) of each interaction model to that of the main effects model. As can be seen in Table 4, the BIC values of the interaction models that included innovativeness (interaction model 1), risk taking (interaction model 3), need for autonomy (interaction model 4), and need for achievement (interaction model 5) were smaller than the BIC value of the main effects model. As can be seen in these models, the interaction effects of innovativeness, risk taking, need for autonomy, and need for achievement with venture life cycle were significantly and positively related to the intercept of number of employees. In addition, the interaction effect of need for achievement with venture life cycle was significantly and negatively related to the slope of number of employees.

**Figure 2:**
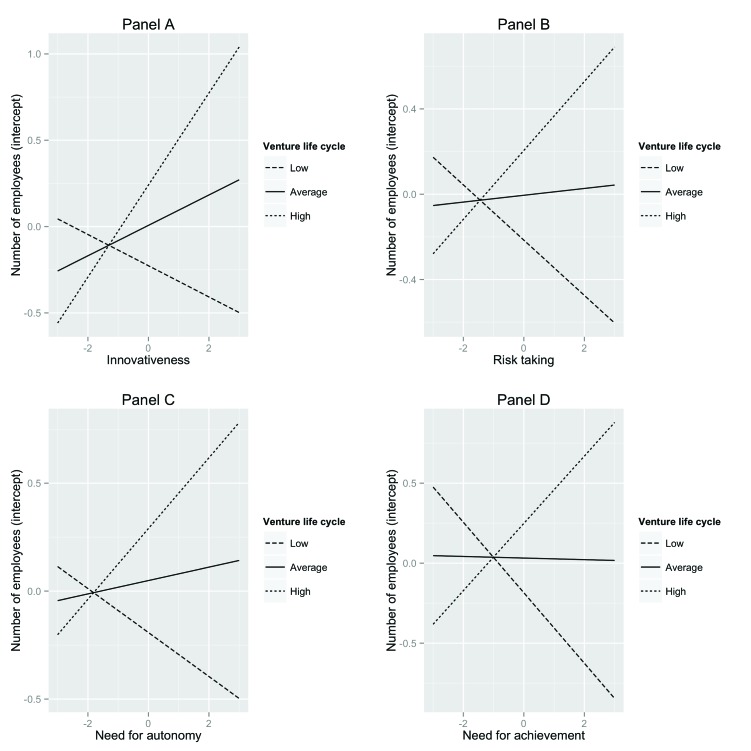
Simple slope plots for interaction effects of venture life cycle and innovativeness (panel A), venture life cycle and risk taking (panel B), venture life cycle and need for achievement (panel C), and venture life cycle and need for achievement (panel D) explaining variance in the intercept of number of employees.

To interpret the four interaction terms that were significantly related to the intercept of number of employees, we again plotted simple slopes for low (1SD below the mean), average, and high (1SD above the mean) values of venture life cycle (see Figure 2). As can be seen in Panel A of Figure 2, there is a significant positive relationship between innovativeness and the intercept of number of employees when venture life cycle is high (*est.* = .27, *t* = 3.73, *p* < .001), and a non-significant relationship when venture life cycle is average (*est.* = .09, *t* = 1.78, *p* = .08) or low (*est.* = ­.09, *t* = ­1.33, *p* = .18). As can be seen in Panel B, there is a positive relationship between risk taking and the intercept of number of employees when venture life cycle is high (*est.* = .16, *t* = 2.94, *p* = .004), a non-significant relationship when venture life cycle is average (*est.* = .02, *t* = .46, *p* = .64), and a significant negative relationship when venture life cycle is low (*est.* = ­.13, *t* = ­2.35, *p* = .02). Similarly, Panel C shows a significant positive relationship between need for autonomy and the intercept of number of employees when venture life cycle was high (*est.* = .16, *t* = 3.86, *p* < .001), a non-significant relationship when venture life cycle was average (*est.* = .03, *t* = 1.35, *p* = .18), and a significant negative relationship when venture life cycle was low (*est.* = ­.10, *t* = ­2.53, *p* = .01). Lastly, Panel D shows a significant positive relationship between need for achievement and the intercept of number of employees when venture life cycle was high (*est.* = .21, *t* = 3.61, *p* < .001), a non-significant relationship when venture life cycle was average (*est.* = ­.01, *t* = ­.13, *p* = .89), and a significant negative relationship when venture life cycle was low (*est.* = ­.22, *t* = ­3.76, *p* < .001). In sum, we could partly confirm Hypothesis 6, as venture life cycle moderated the relationships of four of the five individual EO variables and the intercept of employment.

**Figure 3:**
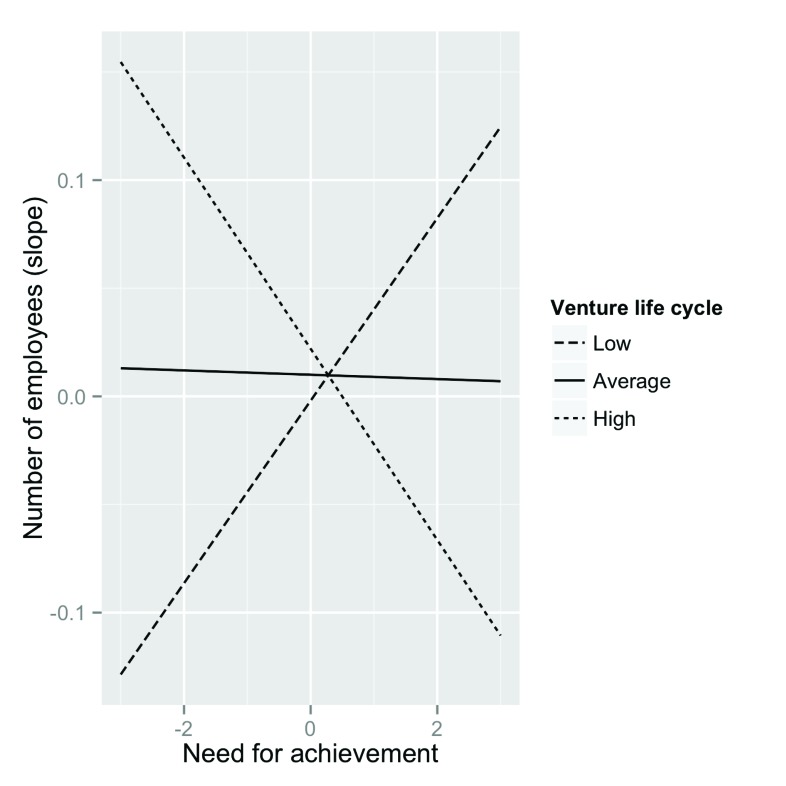
Simple slope plots for interaction effect of venture life cycle and need for achievement explaining variance in the slope of number of employees.

Finally, we plotted simple slopes for low, average, and high values of venture life cycle to interpret the interaction effect between need for achievement and venture life cycle when explaining the slope of revenue. As can be seen in Figure 3, there was a significant negative relationship between need for achievement and the slope of employment when venture life cycle was high (*est.* = ­.04, *t* = ­2.82, *p* = .01), a non-significant relationship when venture life cycle was average (*est.* = ­.001, *t* = ­.10, *p* = .92), and a significant positive relationship when venture life cycle was high (*est.* = .04, *t* = 2.46, *p* = .01). We thus could again partly confirm Hypothesis 6, as venture life cycle moderated the relationships of one of the five individual EO variables and the slope of revenue.

## Discussion

This study is among the first to transfer EO to the individual level. By doing so, we aim to reinvigorate the debate on the role of entrepreneurial personality. Based on literature and previous findings, we hypothesized that the five dimensions of individual EO differentiate between entrepreneurs and non-entrepreneurs, and explain objective entrepreneurial success. Moreover, we included venture life cycle as a moderator in the relationships between individual EO and entrepreneurial success. In particular, we contrasted two perspectives that explained the moderating role of venture life cycle. According to the situation strength perspective, the five individual EO traits ought to be most strongly related to venture performance when venture life cycle is low, because early phases of the venture life cycle can be considered ‘weak’ situations with high uncertainty and ambiguity (Meyer et al., 2010). According to the differentiation perspective, the five individual EO traits ought to be most strongly related to venture performance when venture life cycle is high, because mature venture can use the entrepreneurs’ personality traits as a unique resource to differentiate themselves from similar competitors and, hence, attain a competitive advantage (Wiklund & Shepherd, 2005).

Our findings revealed that three of the five individual EO traits—namely, innovativeness, proactivity, and risk taking—differentiated entrepreneurs from non-entrepreneurs. In line with several prior studies (e.g., Baum & Locke, 2004; Gartner, 1989), we found few statistically significant direct effects of the five individual EO traits on entrepreneurial success. In fact, the only statistically significant direct effect that we observed contradicted our hypotheses, as proactivity was negatively related to the slope of revenue. However, introducing venture life cycle as a moderator helped to explain the absence of direct effects of individual EO, as a clear pattern of interaction effects was found. We found support for a differentiation perspective when explaining differences between ventures in terms of revenue and number of employees, and support for a situation strength perspective when explaining growth in number of employees. Below, we discuss the theoretical implications of these findings.

### Differences between entrepreneurs and non-entrepreneurs

Our first set of hypotheses focused on differences between entrepreneurs and non-entrepreneurs in terms of innovativeness, proactivity, risk taking, need for autonomy, and need for achievement. Our results supported Hypotheses 1a, 2a, and 3a, as entrepreneurs scored higher on innovativeness, proactivity, and risk taking than non-entrepreneurs. These findings can be explained using person-fit theory (Caplan, 1987) and the attraction-selection-attrition model (Schneider, 1987). According to these theories, people self-select an occupation in line with their personality and values. Hence, people with a natural inclination towards innovativeness, proactivity, and risk taking may be more strongly attracted to an entrepreneurial career. Moreover, they may be more likely selected into entrepreneurship, for example because they more easily receive funding from external stakeholders. And finally, these personality traits may help them cope with an entrepreneurial career, which implies that they are more likely to remain an entrepreneur (i.e., attrition). These findings hence confirm prior studies that showed that entrepreneurs scored higher than other occupational groups on innovativeness (e.g., Cromie, 2000; Rauch & Frese, 2007), proactivity (e.g., Crant, 1996; Rauch & Frese, 2007), and risk taking (e.g., Rauch & Frese, 2007; Stewart & Roth, 2001). However, it is important to note that an alternative explanation for these findings cannot be excluded, namely that entrepreneurial experiences may gradually change an entrepreneur’s personality (socialization), leading to personality differences between entrepreneurs and non-entrepreneurs (41). We come back to this alternative explanation when discussing the limitations of the present study.

We could not confirm Hypotheses 4a and 5a. In contrast with prior studies (e.g., Cromie & O’Donaghue, 1992; Rauch & Frese, 2007), we found no differences between entrepreneurs and non-entrepreneurs in terms of need for autonomy and need for achievement. This could be due to the fact that we examined all five individual EO traits together, whereas previous studies often looked at single traits. Not controlling for other traits may lead to the conclusion that there are spurious differences between entrepreneurs and non-entrepreneurs, caused by shared variance between the individual EO traits. Alternatively, need for autonomy and need for achievement may also be important in other occupations. For example, people with a strong need for achievement may pursue a managerial career instead of becoming an entrepreneur (Cromie, 2000). Likewise, people with a strong need for autonomy may be able to satisfy this need as employees inside an organization (Bowen & Lawler III, 2006) or even outside of paid employment as a volunteer (Bidee et al., 2013). Hence, it may be difficult to discern differences between entrepreneurs and non-entrepreneurs on these two traits, because there may be ample variation within the group of non-entrepreneurs. It therefore seems advisable that future studies compare entrepreneurs to people in specific occupations, such as managers or white-collar employees.

### Explaining entrepreneurial success

We could not find support for Hypotheses 1b to 5b, which postulated positive direct effects of the five individual EO traits on entrepreneurial success. Most traits were unrelated to the intercept and slope of revenue and number of employees. One trait formed an exception to this: proactivity was negatively related to the slope of revenue. Entrepreneurs who scored high on proactivity were more likely active in ventures with decreasing revenues over a three-year period, whereas entrepreneurs who scored high on need for autonomy were more likely active in ventures with decreasing numbers of employees over a three-year period. It is possible that high proactive entrepreneurs choose a prospector strategy, meaning that their venture continuously searches the market for new opportunities (Kickul & Gundry, 2002). As a result, they may experience a negative growth in revenue because they continuously invest their resources in new products or services. This could be tested in future studies by relating proactivity to outcomes such as return on investment.

Overall, our findings on the direct effects of the individual EO traits on entrepreneurial success add to the debate on the role of personality in entrepreneurship (Baum & Locke, 2004; Gartner, 1989). Some authors have argued that researchers should abandon research on entrepreneurial personality, because many studies, including ours, fail to find significant relationships with outcomes such as venture performance (Gartner, 1989). However, we need to acknowledge that entrepreneurs operate in complex environments, with many factors that may influence venture performance (Hmieleski & Baron, 2009). For example, Frese (2009) proposed that personality drives entrepreneurial actions, which in turn may lead to success. His model stipulates that these relationships are moderated by various environmental factors, such as environmental dynamism, hostility, industry, and the venture’s life cycle. Failing to take these environmental factors into account may lead to null-effects when relating entrepreneurs’ personality traits to venture performance. We therefore focused on one such environmental factor, namely venture life cycle.

### Moderating role of venture life cycle

In general, our findings support that venture life cycle moderates several relationships between individual EO traits and entrepreneurial success. In particular, innovativeness, risk taking, need for autonomy, and need for achievement were positively related to objective venture performance, but not in all phases of the ventures’ life cycles. A first pattern of findings offered support for the differentiation perspective. Entrepreneurs who scored high on risk taking, need for achievement, and need for autonomy attained higher revenues compared to entrepreneurs who scored low on these traits, when their ventures were in later, mature phases. We argued that these traits may offer a competitive advantage for ventures in later life cycles, because they differentiate the venture from other, similar firms (Wiklund & Shepherd, 2005). Having a strong inclination to take risks may help mature ventures to move into new markets or adopt new strategies, while having a strong need for achievement may help promote a competitive stance towards competitors. Having a strong need for autonomy may stimulate entrepreneurs to keep control over their mature venture, leading to a central vision and strong leadership (Chandler & Hanks, 1998; Lumpkin & Dess, 1996). Entrepreneurs who scored high on innovativeness, risk taking, need for autonomy, and need for achievement had higher numbers of employees in their ventures compared to entrepreneurs who scored low on these traits, when their venture life cycle was high. Being innovative may help mature ventures to move into new markets, requiring new employees (Lumpkin & Dess, 1996). Likewise, an inclination to take risks may help deal with the uncertainty and stress associated with expanding (Lumpkin & Dess, 1996). A strong need for autonomy related to lower numbers of employees when venture life cycle was low, but to higher numbers of employees when venture life cycle is high. However, a mature venture life cycle implies that the venture has grown in size (Rutherford et al., 2003). Hence, entrepreneurs with a low need for autonomy may refrain from moving their venture into later life cycles. A strong need for achievement, finally, was associated with higher amounts of employees when venture life cycle was high, but lower mounts of employees when venture life cycle was low. Possibly, having an achievement oriented entrepreneur in charge may offer a competitive advantage for established firms in later life cycles, because these entrepreneurs have an innate desire to outperform competitors.

A second pattern of findings offered support for a situation strength perspective. Entrepreneurs who scored high on need for autonomy attained higher growth rates in number of employees, compared to entrepreneurs who scored low on this trait, when venture life cycle was low. Need for autonomy may help entrepreneurs grow their firm when the environment is still ambiguous and uncertain (i.e., a ‘weak’ environment; Meyer et al., 2010). As ventures mature, they increasingly become formalized and have cleared norms on how to act, as such diminishing the influence of the entrepreneur’s personality. Interestingly, the relationships became negative when venture life cycle was high. Entrepreneurs scoring high on need for autonomy may choose cost-reducing strategies, such as downsizing and layoffs, to ensure venture survival in these mature life cycle phases.

Overall, our findings clearly show that venture life cycle needs to be taken into account when examining the relationships between individual EO and entrepreneurial success. Novel theoretical models should explicitly address the exact role of each individual EO trait in these different life cycles. As we demonstrated, various perspectives (e.g., situation strength and differentiation perspectives) may apply, depending on the outcome. Future studies need to take into account that while some factors may explain absolute differences in performance between ventures, other factors may explain change in venture performance over time—these factors are not necessarily the same. Moreover, other environmental factors might also be taken into account, such as environmental dynamism, hostility, and industry type (Frese, 2009), to fully understand the influence of entrepreneurial personality on venture performance.

### Limitations and suggestions for future research

Some methodological and conceptual limitations can be discerned in the current study. First, one of the strengths of our study is that we used separate sources for independent and dependent variables—by measuring venture performance with objective financial data—thus decreasing common method bias (Podsakoff et al., 2003). However, our study is cross-sectional, so we cannot make any claim about the causality of the relationships. Personality traits may cause people to become a successful entrepreneur, which would mean that a selection process is at work (Niess & Biesemann, 2014). We took this perspective, following Person-fit theory (Caplan, 1987) and the attraction-selection-attrition model (Schneider, 1987) when developing hypotheses. However, the cross-sectional design of our study does not rule out the presence of a socialization process, meaning that successful entrepreneurial experiences may lead to changes in personality (Niess & Biesemann, 2014). Studies indeed suggest that both processes—selection and socialization—matter, and may even interact (Denissen, Ulferts, Lüdtke, Muck, & Gerstorf, 2014; Roberts, Caspi, & Moffitt, 2003 This interaction, termed the corresponsive principle, indicates that the personality traits that cause a person to self-select into a certain occupation are most likely to change afterwards due to certain work experiences. For example, scoring high on the five individual EO traits may lead a person to pursue a career as an entrepreneur, after which successful entrepreneurial experiences may in turn lead to gradual changes in personality and a further increase in individual EO. We believe that it is an important challenge for future entrepreneurship research to disentangle both processes. This would require longitudinal studies, in which nascent entrepreneurs’ personality traits are measured prior to becoming an entrepreneur and in the years that follow this decision.

Second, we randomly selected entrepreneurs from a database, resulting in a diverse sample. This sample was similar to the general population of entrepreneurs, save for age and gender. However, entrepreneurs constitute a heterogeneous population (Gartner, 1989), so it might prove beneficial for future studies to focus on specific groups of entrepreneurs (e.g. founder-managers of spin-offs) to further control for contextual effects. Alternatively, multilevel studies could help us better understand the interplay between the individual and the environment in entrepreneurship.

Finally, we operationalized individual EO by adapting measures of innovativeness, proactivity, risk taking, need for autonomy, and need for achievement. We revised these measures because they were originally not designed for employees—let alone entrepreneurs. During this process of revision, care was exerted to ensure the final measures would still be reliable and valid, following recommendations by DeVellis (2003). We assessed face validity, item variance, internal reliability and factor structure in a pilot study. In addition, we retested internal reliability and factor structure in the main study. Nonetheless, we believe that these measures can still be improved and need to be further validated. Moreover, one might argue that alternative operationalizations of individual EO are possible. This is especially relevant in case of competitive aggressiveness, which could be operationalized as machiavellism, aggressive dominance, performance goal orientation, or type A behavior (Begley & Boyd, 1987; VandeWalle, Brown, Cron, & Slocum, 1999; Utsch, Rauch, Rothfuss, & Frese, 1999). We chose to operationalize competitive aggressiveness as need for achievement in view of earlier described similarities between both constructs (Kollmann et al., 2007), but we recommend future studies to assess and compare the validity of alternative operationalizations.

### Practical implications

Many practitioners feel that the personality, beliefs, values and behavior of the entrepreneur are of the utmost importance in explaining venture behavior (Chapman, 2000). While the role of personality in entrepreneurship has often been criticized (Gartner, 1989), our results demonstrate that entrepreneurial personality merits attention. First, entrepreneurs differ significantly from the general population on a number of personality traits. A better understanding of the differences between entrepreneurs and non-entrepreneurs can help practitioners and trainers to better detect and support entrepreneurial potential early on. For example, nascent entrepreneurs could be matched to team members based on their personality profiles; an entrepreneur with low innovativeness could be matched to a team-member who scores high on this trait. Second, these traits may help or hinder an entrepreneur in the pursuit of entrepreneurial success. While we found links between individual EO traits and entrepreneurial success, we also found that venture life cycle needs to be taken into account. Early on, one should focus on having an entrepreneur in charge who has a strong need for autonomy, as this could help to grow the venture. Later on, focus should shift towards risk taking, innovativeness, need for autonomy, and need for achievement, as this could help mature ventures to obtain a competitive advantage.

## Conclusion

Our study shows that individual EO—that is, the constellation of five personality traits: innovativeness, proactivity, risk taking, need for achievement, and need for autonomy—offers a meaningful perspective to study and understand entrepreneurship. First, we find that entrepreneurs differ from non-entrepreneurs; as entrepreneurs score higher on innovativeness, risk taking, and proactivity. Second, we establish that entrepreneurs’ individual EO relates to objective venture performance, but only when venture life cycle is taken into account. For ventures in an early life cycle, having an entrepreneur in charge who scores high on need for autonomy may help grow the venture in terms of number of employees. For ventures in a later life cycle, having an entrepreneur in charge who scores high on risk taking, innovativeness, need for achievement, and need for autonomy may help to differentiate from, and thus outperform, competitors.

## Footnotes

We will hence forward speak of need for achievement as the counterpart of competitive aggressiveness on the individual level.
